# Integrative Analysis Reveals Conserved R-Loop Features in Mouse Embryonic Stem Cells

**DOI:** 10.3390/epigenomes10010016

**Published:** 2026-03-02

**Authors:** Ohbeom Kwon, Hyeonwoo La, Seonho Yoo, Hyeonji Lee, Heeji Lee, Hoseong Lim, Chanhyeok Park, Dong Wook Han, Jeong-Tae Do, Hyuk Song, Youngsok Choi, Kwonho Hong

**Affiliations:** 1 Department of Stem Cell and Regenerative Biotechnology, Institute of Advanced Regenerative Science, Konkuk University, Seoul 05029, Republic of Korea; scpzt0311@konkuk.ac.kr (O.K.); roflcopter@konkuk.ac.kr (H.L.); upreference98@naver.com (S.Y.); affogatojoa@konkuk.ac.kr (H.L.); heeji35@konkuk.ac.kr (H.L.); limghtjd@konkuk.ac.kr (H.L.); chpark0729@gmail.com (C.P.); dojt@konkuk.ac.kr (J.-T.D.); songh@konkuk.ac.kr (H.S.); choiys3969@konkuk.ac.kr (Y.C.); 2 School of Life Sciences, Suzhou Medical College of Soochow University, Suzhou 215123, China; dwhan.stem@gmail.com

**Keywords:** R-loop, mouse embryonic stem cell, chromatin states, GC skew, G-quadruplex, transcription regulation, post-transcriptional regulation, RNA metabolism

## Abstract

R-loops, three-stranded nucleic acid structures formed by an RNA-DNA hybrid, have emerged as important regulators of transcription and genome stability. Although advances in high-throughput sequencing have revealed widespread R-loop landscapes, platform-specific biases hinder the identification of conserved R-loops in specific cell types. Mouse embryonic stem cells, which are transcriptionally active, provide an ideal system for investigating the potential roles of stable R-loops in RNA biology. Here, we integrated 13 independent R-loop profiling datasets from four experimental platforms to define 27,950 Common R-loop regions in mouse embryonic stem cells and characterized their chromatin environment and associated biological functions. Common R-loop regions were reproducibly detected across methods and were preferentially localized to promoter-proximal and genic regions enriched in CpG islands. Genes associated with Common R-loops were highly and stably expressed, showing strong functional enrichment in RNA metabolic processes such as mRNA processing, RNA splicing, and ribonucleoprotein complex biogenesis. Chromatin state analysis revealed that Common R-loops are enriched in transcriptionally active and regulatory contexts. Sequence feature analysis further identified GC skew as a prominent signature of Common R-loops, particularly within transcribed chromatin states. Transcription factor motif analyses have identified distinct regulatory environments in Common R-loop regions, including pluripotency-associated OCT4-SOX2-TCF-NANOG motifs in enhancers, CTCF motifs in open chromatin, and YY1 motifs in promoters. Together, this study provides the first integrated analysis of conserved R-loop regions in mouse embryonic stem cells, revealing their preferential localization at regulatory loci linked to RNA metabolism and highlighting R-loops as structural and functional nodes in RNA biology.

## 1. Introduction

R-loops are three-stranded nucleic acid structures in which an RNA transcript hybridizes with its complementary DNA strand, displacing the non-template DNA as a single strand [1,2]. Initially regarded as rare byproducts of transcription, R-loops are now recognized as important regulators of transcription and genome stability [3,4,5]. Dysregulation of R-loop homeostasis has been implicated in diverse pathological contexts, including neurodegenerative disorders, immunodeficiencies, and cancer, often through the disruption of RNA metabolism and transcription-coupled genome maintenance [6,7,8,9,10].

The importance of R-loops in RNA biology lies in their ability to modulate multiple stages of gene expression. They influence transcription initiation and pausing, pre-mRNA processing, RNA stability, and the recruitment of chromatin modifiers [11,12,13,14,15,16,17,18,19]. These functions directly intersect with processes central to both development and disease. For instance, aberrant R-loop accumulation can impair RNA splicing, alter RNA export, and destabilize transcriptional mechanisms linked to tumorigenesis and other RNA-associated disorders [20,21,22,23,24,25,26,27,28,29].

Advances in high-throughput R-loop profiling, including DRIP-seq (DNA-RNA immunoprecipitation), MapR, HBD-seq (Hybrid binding domain), and CUT&Tag, have provided precise, genome-wide R-loop distributions [30,31,32,33,34,35]. However, platform-specific biases generate variability in R-loop peaks, complicating the identification of conserved R-loop regions that represent stable functional features within a given cell type. Previous studies have classified R-loops by their genomic context, such as promoter-paused or elongation-associated, and distinguished constitutive from variable R-loops [12,36]. However, these studies often aggregated heterogeneous datasets from multiple cell types, limiting insights into cell-type-specific and biologically conserved features.

Here, we address these limitations by integrating 13 R-loop datasets from mouse embryonic stem cells (mESCs) and applying stringent criteria to identify R-loop regions consistently detected across multiple profiling strategies. We analyzed chromatin states and sequence features to define the genomic, epigenomic, and regulatory landscapes of conserved R-loop regions. Our findings reveal that Common R-loops are preferentially located in transcriptionally active and regulatory chromatin states. The genes harboring these R-loops are enriched for RNA metabolic pathways. Furthermore, these R-loops intersect with transcription factor networks critical for pluripotency and RNA processing, underscoring their potential roles as structural and functional nodes in RNA biology and disease.

## 2. Results

### 2.1. Integration of R-Loop Datasets Defines Common R-Loop Regions

**Figure 1:**
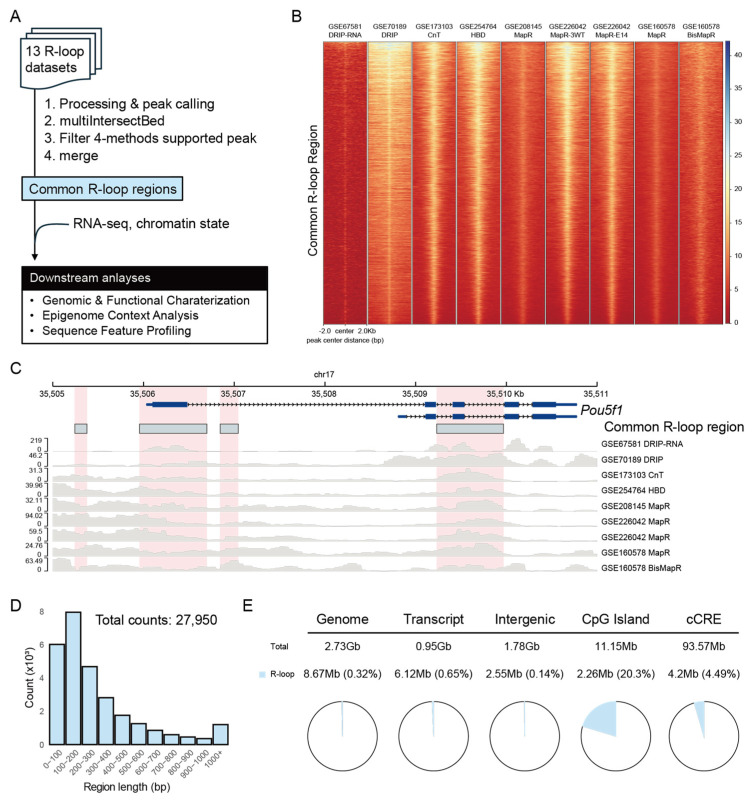
Integration of R-loop profiling datasets defines Common R-loop regions. (**A**) Schematic workflow for defining Common R-loop regions by integrating 13 datasets generated from four R-loop profiling methods. RNA-seq data were used for functional enrichment analysis, and chromatin state data were used for epigenome context profiling. (**B**) R-loop signals within ±2 kb of the center of Common R-loop regions. Each row on the y-axis corresponds to a single Common R-loop region. RPGC (Reads Per Genome Coverage) normalized bigwig files were used for visualization. (**C**) Genome browser snapshot of R-loop datasets in the *Pou5f1* locus, showing that Common R-loop regions (sky blue boxes) are consistently enriched for R-loop signals across methods. (**D**) Length distribution of Common R-loop regions, with the most frequent length range observed between 100 and 200 bp. (**E**) Genomic distribution of the Common R-loop regions, showing preferential enrichment within transcribed and regulatory regions. Transcript regions were defined using UCSC mm10 RefSeq annotations. CpG islands and candidate cCREs were defined using UCSC genome annotations.

To define R-loop regions reproducibly detected across diverse experimental platforms, we integrated 13 R-loop datasets using four methods: DRIP-based approaches, CUT&Tag, HBD-seq, and MapR (Figure 1A). Using a stringent intersection strategy with bedtools multiIntersectBed, we identified the genomic loci supported by at least one dataset from each method and reconstructed the full span of these overlapping peaks. This approach yielded 27,950 method-independent R-loop regions, which we hereafter refer to as Common R-loop regions. Despite platform-specific differences in signal intensity (Figure S1A), these regions showed consistent and robust R-loop enrichment across datasets (Figure 1B). Genome browser views at representative loci (e.g., *Gapdh*, *Actb*, and mESC markers *Pou5f1*, *Nanog*, and *Sox2*) confirmed that multiple independent datasets converged at the same genomic locations, underscoring the reliability of the defined regions (Figure 1C and Figure S1B). Most Common R-loop regions were compact, with 93.5% ranging between 10 and 1000 bp and most frequently between 100 and 200 bp (Figure 1D and Figure S1C–E), which is consistent with the expected physical footprint of R-loops [37,38,39]. Although these regions collectively cover only 0.31% of the mouse genome, they are preferentially enriched within transcribed regions rather than intergenic spaces (Figure 1E). Notably, 20.3% of CpG islands and 4.49% of candidate cis-regulatory elements (cCREs) overlapped with Common R-loop regions, suggesting a potential role for R-loops in gene regulatory mechanisms.

To explore the regulatory potential of these regions, we analyzed their genomic proximity and functional associations using GREAT [40]. Most Common R-loop regions were located within 500 kb of a transcription start site (TSS) and were associated with one or two genes (Figure S2A). Functional enrichment analysis revealed a strong association with post-transcriptional gene regulation, particularly with pathways related to RNA processing, splicing, and translation (Figure S2B). Consistently, Gene Ontology Cellular Component (GOCC) and Molecular Function (GOMF) analyses of Common R-loop–associated genes were enriched for ribonucleoprotein complexes, P-bodies, and RNA-binding activities. The results indicate that Common R-loops are enriched within the putative regulatory elements of genes involved in RNA metabolism.

### 2.2. Common R-Loops Are Preferentially Enriched at Genic Regions

**Figure 2:**
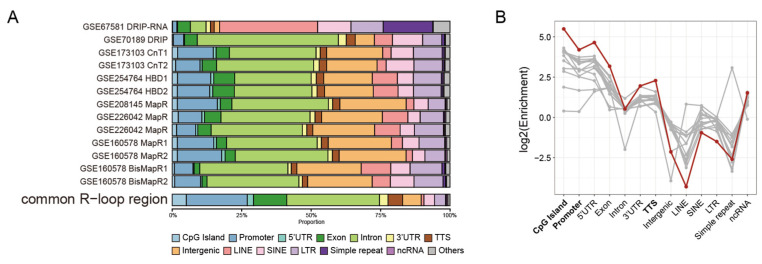
Common R-loops preferentially enriched at genic regions. (**A**) Genomic annotation of Common R-loop regions compared with individual datasets. The category “Others” represents low-frequency annotations combined into a single group, including snoRNAs, DNA elements, and other minor categories. (**B**) Genomic feature enrichment of individual R-loop peaks (gray) and Common R-loop regions (red). Enrichment was calculated by comparing the observed distribution of annotations with their expected genomic distribution. CpG islands, promoters, 5′UTRs, exons and TTSs are strongly enriched, indicating that Common R-loop regions preferentially localize within genic regions. LINE, long interspersed nuclear element; SINE, short interspersed nuclear element; LTR, long terminal repeat; ncRNA, non-coding RNA.

To characterize the genomic distribution of Common R-loops, we compared their annotation profiles with those of individual datasets using HOMER. Common R-loop regions were markedly enriched at CpG islands, promoters, 5′UTR, exons, and transcription termination sites (TTSs), with enrichment exceeding that of individual peaks (Figure 2A,B). This indicates that R-loops are preferentially retained in canonical regulatory elements and gene regions, consistent with their established roles in transcriptional regulation. Because annotation criteria differ among tools, we cross-validated the results using ChIPseeker, which defines promoter regions more broadly and separates the first exon and intron from the rest. While HOMER annotated 21.9% of the regions as promoter-TSS, ChIPseeker classified 64.9% as promoters (Figure S3A–C). More than half of the introns (57.3%) and exons (68.3%) annotated by HOMER corresponded to the promoter regions defined by ChIPseeker (Figure S3E,F). Thus, many Common R-loop regions are localized to areas proximal to TSSs. HOMER also assigns a larger fraction of R-loops to TTS regions because of its broader definition of gene-end boundaries. UpSet analysis confirmed that many of these TTS-labeled regions overlapped with the promoters or gene body elements (Figure S3D). Taken together, these results demonstrate that Common R-loops predominantly reside within genic and promoter-proximal regulatory regions, serving as genomic landmarks critical for transcriptional control and gene regulation.

### 2.3. Common R-Loops Are Associated with Highly Expressed RNA-Regulatory Genes

**Figure 3:**
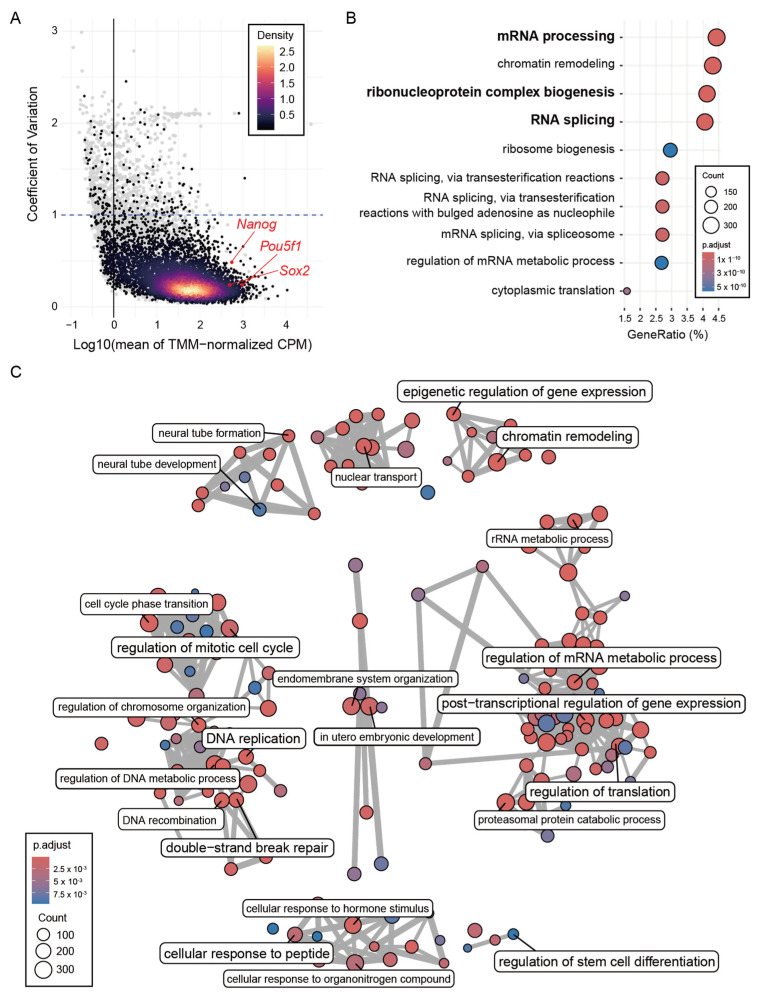
Common R-loops are associated with highly expressed RNA-regulatory genes. (**A**) Mean expression (log10 TMM-normalized CPM) versus coefficient of variation (CV) of 14,655 genes (gray dots). Genes associated with Common R-loops (*n* = 7757) are densely distributed in the high expression and low variability region (gradient dots). Representative pluripotency markers (*Pou5f1*, *Sox2*, and *Nanog*) are indicated. TMM, trimmed mean of M-values; CPM, count per million. (**B**) Top 10 GOBP terms for Common R-loop-associated genes. (**C**) Semantic similarity–based network clustering of 166 enriched GOBP terms. The largest cluster consists predominantly of RNA-related processes, while other clusters include pathways related to essential cellular processes.

Given that Common R-loop regions are predominantly localized to promoter-proximal regions, we next examined the genes associated with these sites and their biological functions. To do this, we analyzed 10 RNA-seq datasets and identified 14,655 genes expressed in mESCs. Based on TSS proximity, 7757 genes were classified as Common R-loop-associated genes. These R-loop-associated genes showed high and stable expression in mESCs (Figure 3A). GO over-representation analysis of R-loop-associated genes identified 166 statistically significant Gene Ontology Biological Process (GOBP) terms. The most enriched biological processes involved post-transcriptional regulatory pathways, such as mRNA processing, RNA splicing, and ribonucleoprotein complex biogenesis (Figure 3B and Figure S4A). Processes essential for cellular maintenance were also identified, such as chromatin remodelling, DNA replication and repair, cell cycle regulation, protein degradation, and signal transduction (Figure S4B). To group these 166 GOBP terms into broader categories, we performed a semantic similarity-based network analysis, which identified eight functional clusters (Figure 3C and Figure S4C). Cluster 1, the largest and most cohesive cluster, was centered on RNA metabolism and was subdivided into modules related to post-transcriptional regulation, ribosome biogenesis, and protein turnover. Other clusters encompassed processes such as cell cycle regulation, DNA replication, chromatin regulation, and signaling pathways, underscoring the broader functional landscape of R-loop-associated genes. These trends were further supported by GOCC and GOMF analyses. Common R-loop-associated genes were significantly enriched for ribonucleoprotein structures, such as the spliceosome, nuclear speckles, and P-bodies (Figure S5). Functionally, these genes were characterized by mRNA binding, ribonucleoprotein complex binding and catalytic activity, acting on a nucleic acid (Figure S6). Cross-validation using DAVID confirmed similar enrichment patterns, particularly for RNA-related functions (Table S2). Collectively, Common R-loops are significantly enriched at genes orchestrating post-transcriptional regulation.

### 2.4. Common R-Loops Exhibit Uniform Positional Patterns Independent of Gene Function

Our GO analysis demonstrated that Common R-loop-associated genes cluster into distinct functional categories. This finding prompted us to investigate whether the genomic positioning of R-loops also differs between these functional groups. We next assessed whether R-loop localization is dictated by specific gene function or if it occurs as a structural by-product of transcription [3]. Using the functional clusters derived from the GOBP network analysis, we compared the distribution of Common R-loops across 11 distinct clusters (Figure S7A). As expected, the positional profiles of the Common R-loops were highly consistent across all functional categories. This uniformity suggests that R-loop localization is not dictated by gene function but is instead governed by general determinants of transcriptional activity and local chromatin structure. This observation supports the view that R-loops form opportunistically during transcription and are stabilized in specific chromatin and sequence contexts [41,42]. To further investigate this, we explored the chromatin state and sequence-level features of Common R-loop regions.

### 2.5. Common R-Loops Are Enriched in Transcriptionally Active and Regulatory Chromatin States

**Figure 4:**
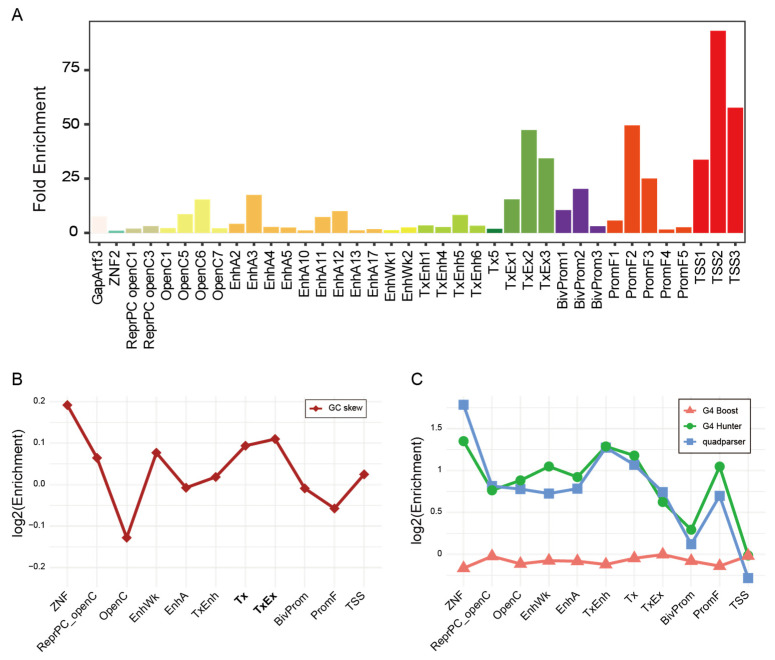
Chromatin state and sequence features define the landscape of Common R-loops. (**A**) The 38 significantly enriched chromatin states. (**B**) Log2-transformed enrichment of mean absolute GC skew across chromatin states. Absolute skew values were calculated within 200 bp windows. (**C**) Log2-transformed enrichment of predicted G4 structures across chromatin states. Each line represents results from three distinct methods: G4Boost (red triangle), G4Hunter (green circle), and quadparser (blue square). For (**B**,**C**), enrichment scores were defined as log2 (mean_R-loop_/mean_background_). Statistical significance was evaluated using a permutation-based subsampling approach (*n* = 10,000).

Building on our observation that R-loop positioning does not depend on gene function, we next investigated their localization in relation to the local chromatin environment. Using a universal chromatin state model that integrates 901 mouse epigenomic datasets [43], we assessed the enrichment of Common R-loop regions across 100 chromatin states. Among these, 38 states were significantly enriched, spanning 12 categories: TSS, Promoter Flank (PromF), Bivalent Promoter (BivProm), Transcription and Exon (TxEx), Transcription (Tx), Transcribed Enhancer (TxEnh), Weak Enhancer (EnhWk), Active Enhancer (EnhA), Open Chromatin (OpenC), Polycomb Repressed and Open Chromatin (ReprPC openC), Zinc Finger Genes (ZNF), and Assembly Gaps and Artifacts (GapArtf) (Figure 4A and Figure S8A). The TSS and PromF states displayed the highest enrichment, followed by TxEx, EnhA, and OpenC, which is consistent with our earlier genomic annotations. These results indicate that Common R-loops are preferentially located within transcriptionally active or regulatory chromatin contexts.

### 2.6. GC Skew as a Sequence Signature of R-Loops in Transcribed Chromatin States

To define chromatin state-specific R-loop regions, the mouse genome was partitioned into 200 bp windows and a ≥50% overlap criterion was applied based on coverage optimization (Figure S8). Previous studies have linked both GC skew and G4 to R-loop formation [42,44]. We examined which feature more closely matches the sequence signature of R-loop regions within each chromatin state. Given the correlation between GC skew and G4 motif density, complementary algorithms were applied to distinguish GC skew from G4 motif enrichment and predicted G4 folding propensity. Absolute GC skew was computed per 200-bp chromatin state window, and Common R-loop regions were compared with state-matched ex-R-loop backgrounds. GC skew was significantly higher in Common R-loop regions across multiple states, including EnhWk, Tx, TxEx, and TSS states (Figure S9B), with the strongest enrichment in the transcribed chromatin states Tx and TxEx (Figure 4B). Next, G4 motif enrichment and predicted G4 structure were evaluated using the same state-stratified approach as for GC skew. Quadparser and G4Hunter identified strong enrichment of putative G4 motifs in R-loop regions, most prominently in TxEnh, Tx, and ZNF states (Figure 4C and Figure S9C,D). However, G4 motif enrichment alone is insufficient to infer structural folding. Therefore, G4Boost, a machine learning–based predictor trained on G4-seq data, was used to estimate G4 folding propensity. Contrary to the motif enrichment results, a comparable predicted G4 structure was observed in Common R-loop regions and state-matched background regions (Figure 4C and Figure S9E). Based on the G4Boost results, we hypothesized that GC skew provides a stronger sequence signature for R-loop localization than predicted G4 folding. Therefore, the hypothesis was tested using a Random Forest permutation importance analysis, in which feature importance was quantified as the mean decrease in AUC upon permutation of each feature (Figure S11). GC skew was the most informative feature for distinguishing Common R-loop regions from matched background (mean decrease in AUC = 0.0737), whereas the G4Boost-derived predicted G4 folding metric was minimally informative (0.0038). GC skew also remained more informative than the total G4 motif count. Together, these results indicate that GC skew is a more robust sequence signature of R-loop localization in mESCs than predicted G4 structure.

### 2.7. Chromatin State-Dependent Motif Enrichment Delineates the Regulatory Context of Common R-Loop Regions

**Figure 5:**
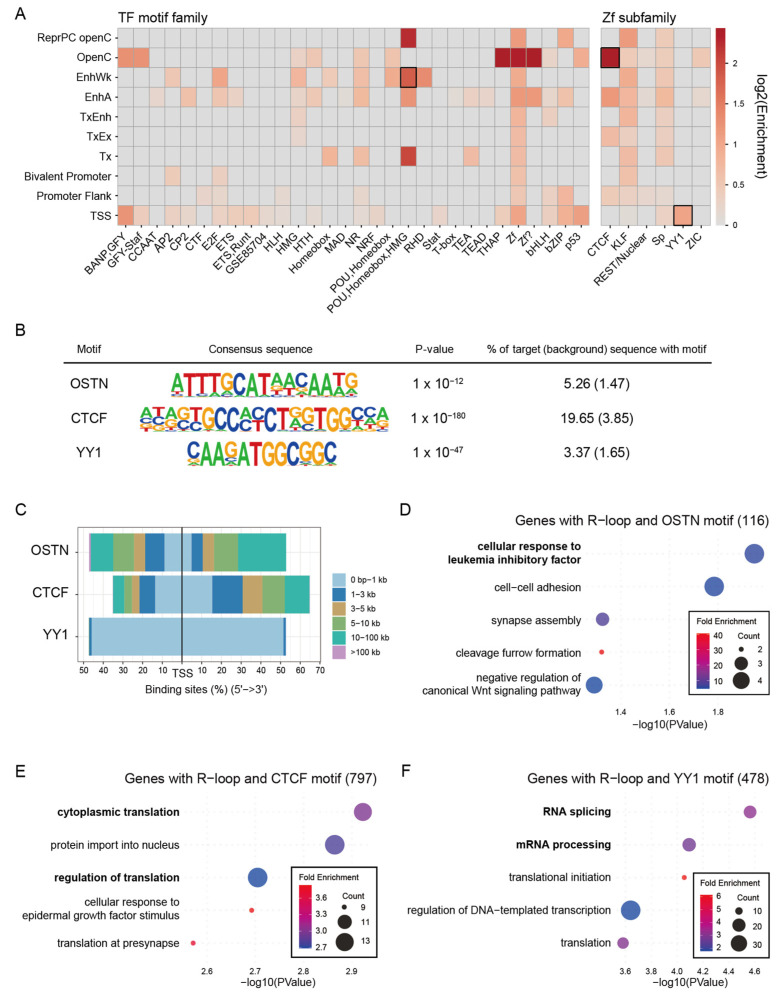
Chromatin state-specific transcription factor motif patterns reveal the regulatory landscape of Common R-loop regions. (**A**) Heatmap displaying the log2-transformed enrichment of TF motif families across different chromatin states. Black boxes highlight three representative motifs (OSTN, CTCF, and YY1) selected for further characterization. (**B**) Detailed statistics for the representative motifs highlighted in (**A**). The table lists the consensus sequence, statistical significance (*p*-value), and the percentage of motif occurrence in the target (R-loop) versus background sequences. (**C**) Genomic distribution of the identified TF motif loci relative to the TSS. The bar chart shows the proportion of motifs located within specific distance intervals. (**D**–**F**) GOBP enrichment analysis for genes proximal to R-loop regions containing (**D**) OSTN, (**E**) CTCF, or (**F**) YY1 motifs. In these dot plots, the x-axis represents the statistical significance (−log_10_ *p*-value), the dot size indicates the number of genes (Count) associated with each term, and the dot color corresponds to the fold enrichment calculated via clusterProfiler.

To investigate the regulatory environment of Common R-loop regions, transcription factor (TF) motif enrichment was analyzed across chromatin states. Distinct chromatin state–specific motif patterns were observed (Figure 5A and Figure S10). Notably, the POU-Homeobox-HMG motifs, corresponding to the core pluripotency factors OCT4, SOX2, TCF, and NANOG (OSTN), were highly enriched in the EnhA, EnhWk, ReprPC openC, and Tx states. These results suggest that R-loops may cooperate with stem cell master transcription factors at distal regulatory regions. Meanwhile, zinc finger (Zf) motifs were widespread across chromatin states. However, unique chromatin-state preferences became apparent after classification into six subclasses. CTCF motifs were enriched in the OpenC, TxEx, EnhA, and PromF states, consistent with the established roles of CTCF in chromatin insulation and enhancer-promoter interactions. A previous study has reported that R-loops are enriched at CTCF binding regions, and our results support these findings [44]. YY1 motifs were enriched in the TSS state, suggesting a promoter-specific regulatory role with R-loops. Notably, YY1 has been reported to bind G4 structures, which often co-occur with R-loops, to mediate enhancer-promoter looping [45,46]. To further explore the functional relevance of these motifs, GO analysis was performed on genes proximal to R-loop regions containing specific TF motifs. Genes associated with both OSTN motifs and Common R-loops were enriched for cellular response to leukemia inhibitory factor, consistent with their role in maintaining pluripotency [47,48] (Figure 5D). Genes associated with CTCF motifs and Common R-loops were enriched for cytoplasmic translation (Figure 5E), whereas genes with YY1 motifs and Common R-loops were enriched for RNA splicing and mRNA processing (Figure 5F). Collectively, these results indicate that R-loops, in conjunction with specific TF motifs, occupy distinct positional and functional niches: OSTN motifs at enhancers are linked to pluripotency, CTCF motifs in open chromatin are linked to translation, and YY1 motifs at promoters are linked to RNA processing.

## 3. Discussion

By integrating multi-platform R-loop profiling datasets from mESCs, we generated high-confidence Common R-loop regions and systematically characterized their genomic distribution, chromatin environment and functional associations. This resource provides a robust reference for reproducible R-loop features in mESCs and offers insights into how R-loops integrate into RNA biology. Defining Common R-loops by cross-dataset concordance necessarily excludes non-consensus peaks, which are heterogeneous and may reflect platform-specific signals and variability due to cell line and experimental differences. Expanded, platform-stratified datasets with controlled designs will be required to resolve their distinct features and regulatory roles in mESCs.

Our findings reinforce the concept that R-loops preferentially localize to promoter-proximal and genic regions enriched in CpG islands, consistent with established roles in transcriptional regulation. Genes associated with Common R-loops were highly and stably expressed and were enriched for RNA metabolism pathways, particularly in mRNA processing, splicing, and ribonucleoprotein complex assembly. However, functional association should be distinguished from direct causality. Although R-loop–associated regions are enriched near genes involved in RNA processing, the enrichment does not establish a direct mechanistic role for the R-loop structures. Although our analyses focused on the functional annotation of R-loop–associated genes rather than physical interactions with RNA processing proteins, independent experimental studies have reported direct associations between R-loops and RNA processing machinery. For example, proximity proteomics has identified enrichment of splicing factors, mRNA export factors, and 3′–end processing proteins near R-loops, and DEAD-box helicases have been shown to bind R-loops and regulate rRNA processing [49,50,51]. In addition, the splicing factor XAB2 has been reported to bind R-loops and modulate intron retention and genomic stability [20], while RNA-processing complexes such as splicing proteins and the TREX complex can limit R-loop accumulation and promote mRNA export [6]. Together, these observations support the possibility that R-loops can provide interaction interfaces for RNA-processing factors and, in specific contexts, influence downstream RNA processing in a transcription–coupled manner.

Chromatin state-aware analyses revealed that Common R-loops are most enriched in transcriptionally active and regulatory chromatin states, including transcription start sites, promoter flanks, and active enhancers, which are frequently marked by H3K4me3 and H3K27ac. Such environments may favor R-loop persistence by supporting robust transcription initiation and elongation and by maintaining accessible DNA templates. At the sequence level, GC skew was significantly higher in Common R-loop regions, particularly in transcriptionally active chromatin states. Putative G4 motifs (Quadparser and G4Hunter) were also significantly more abundant in Common R-loop regions. In contrast, the predicted G4 structure count (G4Boost) was comparable between Common R-loop and background loci. Consistent with these comparisons, Random Forest permutation importance analysis identified GC skew as the most important feature for distinguishing R-loop regions from background, with G4 motif counts providing weaker importance and G4 structure counts contributing minimally. Together, these results suggest that Common R-loops tend to occur at G-rich regions containing G4 motifs. High GC skew likely provides a permissive thermodynamic sequence context that favors RNA:DNA hybridization [30,42]. Accordingly, GC-skewed sequences may favor R-loop formation, producing a displaced G-rich single-stranded DNA. G4 folding could then occur on this displaced strand and potentially stabilize the resulting structure, rather than initiating R-loop formation.

A TF motif enrichment analysis suggests that Common R-loop regions may reflect both cell-type–specific and broadly conserved regulatory contexts. In mESCs, enhancer-like R-loops are enriched for pluripotency-associated factor motifs, linking the sites to stem cell identity programs. Therefore, further studies will be required to determine whether enhancer-resident R-loops causally regulate pluripotency maintenance or lineage commitment. On the other hand, enrichment of motifs for general transcription factors including CTCF and YY1 may reflect a more broadly conserved regulatory mechanism. CTCF-associated R-loops were enriched at open chromatin and linked to translation-related genes, whereas YY1-associated R-loops were enriched at TSSs and associated with RNA splicing and mRNA processing. Given the known function of CTCF and YY1 in chromatin organization, the results raise the possibility that these R-loops coordinate post-transcriptional pathways across various biological contexts [44,52,53]. In addition, disruption of R-loop regulation at the conserved loci could affect essential RNA processing pathways and potentially contribute to RNA metabolism disorders, cancer, and other transcription-associated diseases [41,54,55,56]. For example, altered YY1-associated R-loops could directly contribute to the aberrant splicing patterns frequently observed in cancer and neurodegenerative disorders. Defects in CTCF-associated R-loops may perturb translational control required for cellular homeostasis.

**Figure 6:**
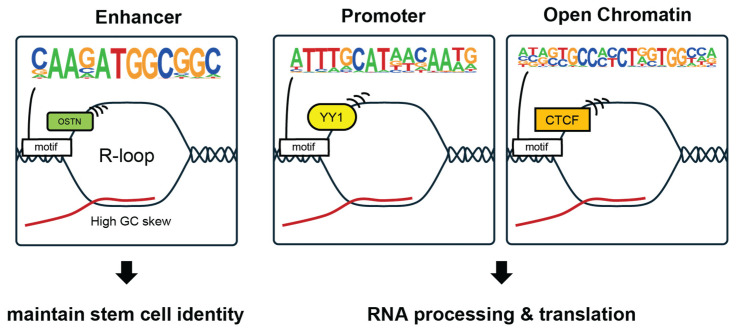
Proposed model of Common R-loop function in mESCs. A proposed model integrating chromatin context, GC skew, and transcription factor motif enrichments at the Common R-loop regions. Common R-loops are broadly detected in accessible open chromatin. Enhancer-associated Common R-loop regions show higher GC skew and enrichment of the OSTN motif. Promoter-proximal and other accessible Common R-loop regions are enriched for motifs of transcription factors such as YY1 and CTCF and are associated with genes involved in RNA processing and translation.

In summary, we propose a model for the Common R-loop function in mESCs (Figure 6). In the model, Common R-loops are enriched in accessible chromatin with distinct transcription factor motif signatures, associating enhancer-linked regions with stem cell identity programs and promoter/open-chromatin regions with RNA processing and translation.

## 4. Materials and Methods

### 4.1. Curation of R-Loop Datasets

Public R-loop profiling datasets for mESCs were collected by searching the GEO DataSets database with the keywords “R-loop” and “RNA-DNA hybrid,” filtering for *Mus musculus* data. This search yielded 78 R-loop and 35 RNA-DNA hybrid datasets. Of these, 22 datasets (13 R-loop, 4 RNase-treated, and 4 MNase) from seven studies were selected (Table S1) [34,35,44,57,58,59,60]. The selected R-loop profiling methods included DRIP-seq, DRIP-RNA-seq, MapR, BisMapR, HBD-seq, and CUT&Tag. Datasets were categorized by R-loop probe type (S9.6 or dRNaseH) and the timing of R-loop detection (ex vivo or in situ).

### 4.2. Public Data Processing

The MapR, BisMapR, HBD-seq, CUT&Tag, and DRIP-seq datasets were processed as follows: reads were trimmed using fastp (v0.23.4) [61,62] and aligned to the mouse genome (mm10) using bowtie2 (v2.5.4) [63]. Raw BAM files were filtered using samtools (v1.19.2) [64] to remove low-quality and unmapped reads (flag 0 × 4), retaining only properly paired reads (flag 0 × 2) with a MAPQ ≥ 10. To minimize PCR duplicates, BAM files were processed with samtools fixmate, coordinate-sorted, and deduplicated using samtools markdup -r. To eliminate mitochondrial DNA, reads aligned to chromosome chrM were removed using samtools view. Reads mapped to the ENCODE mm10 blacklist regions were removed using bedtools intersect (v2.31.1) [65].

The DRIP-RNAseq datasets were processed as follows: reads were trimmed using fastp and aligned to the mouse genome (mm10) using STAR aligner (v2.7.11b) [66]. Raw BAM files were filtered using samtools to retain only reads with MAPQ ≥ 10, excluding unmapped reads. Mitochondrial reads (chrM) were removed. Blacklist filtering was performed using bedtools intersect.

Coverage tracks were generated using deepTools (v3.5.5) bamCoverage [67]. Peak calling was performed using MACS3 (v3.0.2) callpeak [68].

RNA-seq datasets were processed as follows: reads were trimmed using fastp and aligned to the mouse genome (mm10) using the STAR aligner. Read counts per gene were quantified using featureCounts (v2.1.1) [69]. Lowly expressed genes were filtered by calculating counts per million (CPM), retaining genes with CPM ≥ 1 in at least one sample. TMM normalization was applied to correct for library size differences across samples. After filtering and normalization, a final set of expressed genes was established for use as the background gene set for subsequent enrichment analyses.

### 4.3. Identification of Common R-Loop Regions

To define consensus R-loop regions reproducibly detected across different profiling methods, narrowPeak files were processed using bedtools multiIntersectBed (v2.31.1). Regions were retained if at least one dataset from each experimental group (CUT&Tag, HBD, DRIP, or MapR) exhibited a peak. To preserve the biological extent of the observed peaks and avoid the reduction in peak size that can occur with intersection operations, we retrieved the full span of the original peaks that contribute to each intersection. Adjacent or overlapping peaks were merged using bedtools merge. The final merged regions were designated as Common R-loop regions.

### 4.4. Gene Annotation and Functional Enrichment Analysis

Common R-loop regions were annotated to the nearest genes using ChIPseeker (v1.44.0) [70] with the mm10 mouse genome annotation. Gene Ontology enrichment analysis was performed using clusterProfiler (v4.16.0) [71] with all expressed genes from the RNA-seq data as the background. GO terms with *p*-value < 0.01 and *q*-value < 0.05 were considered statistically significant. Redundancy reduction and clustering were performed using enrichment map-based approaches. DAVID [72,73] was used for the cross-validation.

### 4.5. Chromatin State Annotation

Enrichment of Common R-loop regions across chromatin states was assessed using fold enrichment values calculated via ChromHMM (v1.26) [74] based on universal chromatin state annotations [43]. Fold enrichment was defined as (C/A)/(B/D), where:

A = total bases assigned to a chromatin state;

B = total bases covered by Common R-loop regions;

C = overlap bases between the chromatin state and Common R-loop regions;

D = total genome size.

### 4.6. Construction of Chromatin State-Specific R-Loop Regions

The mouse genome was partitioned into non-overlapping 200 bp windows based on the universal chromatin state annotation. Contiguous segments of the same state were divided into uniform windows. Windows overlapping Common R-loop regions were identified using bedtools intersect. Several overlap thresholds (-f options: no threshold, 0.2, 0.5, and 0.8) were tested; the -f 0.5 threshold was selected based on the coverage and chromatin state representation (Figure S8).

### 4.7. Nucleotide Skewness Analysis

GC skew [(G − C)/(G + C)] and AT skew [(A − T)/(A + T)] were calculated for each 200 bp window. Since strand information was not available, absolute skew values were used. Skewness was compared between R-loop-rich regions and ex-R-loop background regions within each chromatin state.

### 4.8. G-Quadruplex Motif Analysis

Canonical G-quadruplex (G4) motifs were identified using a regular expression-based Quadparser (G_3+_N_1–7_G_3+_N_1–7_G_3+_N_1–7_G_3+_) [75]. G4Hunter was applied to score G4-forming potential; scores ≥ 1.2 were considered G4-positive [76]. G4Boost, a machine-learning model trained on G4-seq data, was used to predict G4 formation. The frequency and score of the G4 motifs calculated using each method were compared between R-loop-rich regions and ex-R-loop backgrounds [77].

### 4.9. Transcription Factor Motif Enrichment Analysis

Motif enrichment was analyzed using HOMER (v5.1) [78]. R-loop-rich regions were used as the input, and the corresponding ex-R-loop segments were used as the background. *p*-values, *q*-values, and fold enrichment were computed using HOMER with hypergeometric/binomial models.

### 4.10. Statistical Analysis

For nucleotide skewness and G4 motif analyses, statistical significance was evaluated using a permutation-based subsampling approach. Within each chromatin state, the same number of background regions as the input was randomly sampled 10,000 times to generate a null distribution. The empirical *p*-values were calculated based on the proportion of background means greater than or equal to the observed input mean. *Z*-scores were computed for a standardized effect size. The enrichment score was calculated using the following equation: $log2\left(\frac{mean_{R-loop}}{mean_{background}}\right)$. Statistical significance was determined internally for HOMER-based motif enrichment.

## 5. Conclusions

By systematically integrating multi-platform R-loop profiling datasets from mESCs, we identified a high-confidence set of conserved R-loop regions. These Common R-loops are enriched at promoter-proximal and genic loci, particularly within genes involved in RNA metabolism. Their localization within transcriptionally active chromatin states and association with specific transcription factor networks, such as OCT4-SOX2-TCF-NANOG, CTCF, and YY1, support a model in which conserved R-loops function as structural hubs that bridge transcriptional and post-transcriptional regulation. The identification of GC skew as a robust sequence signature, distinct from G4 structure, refines our understanding of R-loop stability in active chromatin environments. This work establishes a foundational resource for investigating R-loop function in stem cell biology and provides a framework for identifying conserved regulatory R-loops in other cell types and disease states. The reproducibility of these R-loop sites across platforms suggests they may serve as robust biomarkers for R-loop dysregulation, while their functional enrichment at RNA processing loci highlights them as potential therapeutic targets for diseases involving aberrant RNA metabolism.

## Data Availability

The original data presented in the study are openly available in GSE70189, GSE67581, GSE173103, GSE208145, GSE226042, GSE160578, GSE254764, GSE262195, GSE232524, GSE226214, and GSE254763.

## Supplementary Materials

The following supporting information can be downloaded at: https://www.mdpi.com/article/10.3390/epigenomes10010016/s1.

## Abbreviations

The following abbreviations are used in this manuscript:

mESC	Mouse embryonic stem cell
DRIP	DNA-RNA immunoprecipitation
HBD	Hybrid binding domain
GOBP	Gene ontology biological process
GOCC	Gene ontology cellular component
GOMF	Gene ontology molecular function
cCRE	Candidate cis-regulatory element
TTS	Transcription termination site
LINE	Long interspersed nuclear element
SINE	Short interspersed nuclear element
LTR	Long terminal repeat
ncRNA	Non-coding RNA
TSS	Transcription start site
PromF	Promoter flank
BivProm	Bivalent promoter
TxEx	Transcription and exon
Tx	Transcription
TxEnh	Transcribed enhancer
EnhA	Active enhancer
OpenC	Open chromatin
ReprPC openC	Polycomb repressed and open chromatin
ZNF	Zinc finger genes
GapArtf	Assembly gaps and artifacts
OSTN	OCT4-SOX2-TCF-NANOG
NR	Nuclear receptor
Zf	Zinc finger
