# Supplementary material for: Integrative Analysis Reveals Conserved R-Loop Features in Mouse Embryonic Stem Cells
Source: Epigenomes. 2026 Mar 2;10(1):16. doi: 10.3390/epigenomes10010016 (PMC13025856; doi:10.3390/epigenomes10010016)
Supplement: Supplementary file 1 [file epigenomes-10-00016-s001.zip › supple figure legend.pdf]

Fig. S1. Identification of common R-loop region

(**A**) Signal profiles of individual R-loop datasets in each peak and common R-loop region. (**B**) Representative genome browser snapshots showing R-loop signal alignment across datasets at Gapdh, Actb, Nanog, and Sox2 loci. (**C**) Length distribution histogram of common R-loop regions. (**D**) Violin and density plots of R-loop region lengths. The purple area indicates the inner range excluding outliers. (**E**) Proportion of common R-loop regions falling within 10 to 1,000 bp in length.

Fig. S2. GREAT analysis of common R-loop region

(**A**) Histograms showing the number of genes associated per R-loop region (left) and the distribution of distances from each region to the nearest transcription start site (center and right). (**B**) Top enriched GOBP, GOCC, GOMF terms for genes nearest to common R-loop regions.

Fig. S3. Comparison of genomic feature annotation

(**A**) Genomic distribution of common R-loop regions (ChIPseeker annotation). (**B**) Genomic distribution of common R-loop regions (HOMER annotation). (**C**) Comparison of each annotation ratio between ChIPseeker and HOMER. (**D**) Upset plot of ChIPseeker annotation results. (**E**) Overlap between the regions annotated promoter with ChIPseeker and regions annotated exon with HOMER. (**F**) Overlap between the regions annotated promoter with ChIPseeker and regions annotated intron with HOMER.

Fig. S4. GOBP enrichment analysis of genes proximal to common R-loop region
(**A**) Bar plot showing the top 10 enriched GOBP terms ranked by adjusted p-values. (**B**) Dot plot displaying the top 30 enriched GOBP terms ranked by gene ratio. (**C**) Semantic similarity–based network analysis of 166 significantly enriched GOBP terms. Nodes are grouped and color-coded by cluster assignment, with each color indicating a distinct functional module.

Fig. S5. GOCC enrichment analysis of genes proximal to common R-loop region

(**A**) Bar plot showing the top 10 enriched GOCC terms ranked by adjusted p-values. (**B**) Dot plot displaying the top 30 enriched GOCC terms ranked by gene ratio. (**C**) Semantic similarity–based network analysis of 67 GOCC terms.

Fig. S6. GOMF enrichment analysis of genes proximal to common R-loop region

(**A**) Bar plot showing the top 10 enriched GOMF terms ranked by adjusted p-values. (**B**) Dot plot displaying the top 30 enriched GOMF terms ranked by gene ratio. (**C**) Semantic similarity–based network analysis of 46 GOMF terms.

Fig. S7. Common R-loops exhibit uniform positional patterns independent of gene function

(**A**) Normalized fraction of genes harboring R-loops within each genomic annotation category across gene clusters.

Fig. S8. Chromatin state-based characterization of common R-loop region.
(**A**) Enrichment of common R-loop region across chromatin state defined by ChromHMM. (**B**) Proportion of common R-loop region overlapping with each chromatin state, evaluated at different overlap threshold. (**C**) Stacked bar plot showing the chromatin state composition of R-loop region at each overlap threshold. (**D**) Genomic coverage comparison between common R-loop region (yellow) and the union of chromatin state regions overlapping at each threshold (black).

Fig. S9. Summary of GC skew and G4 motif analysis across chromatin state.
(**A**) Schematic of analysis workflow. (**B)** Comparison of GC skew. (**C**) Comparison of G4 motif counted by quadparser between common R-loop region (sky blue) and background means (light gray). (**D**) Comparison of G4 motif scores (left) and counts (right) calculated by G4Hunter. (**E**) Comparison of G4 motif folding probabilities (left) and counts (right) predicted by G4Boost.

Fig. S10. Transcription factor motif analysis of common R-loop region across chromatin state.

(**A**) Dot plot showing the top 10 enriched motifs ranked by p-values across chromatin state. Dot size represents the percentage of target sequences containing each motif.
