# Supplementary figures and images for: Integrative Analysis Reveals Conserved R-Loop Features in Mouse Embryonic Stem Cells
Source: Epigenomes. 2026 Mar 2;10(1):16. doi: 10.3390/epigenomes10010016 (PMC13025856; doi:10.3390/epigenomes10010016)

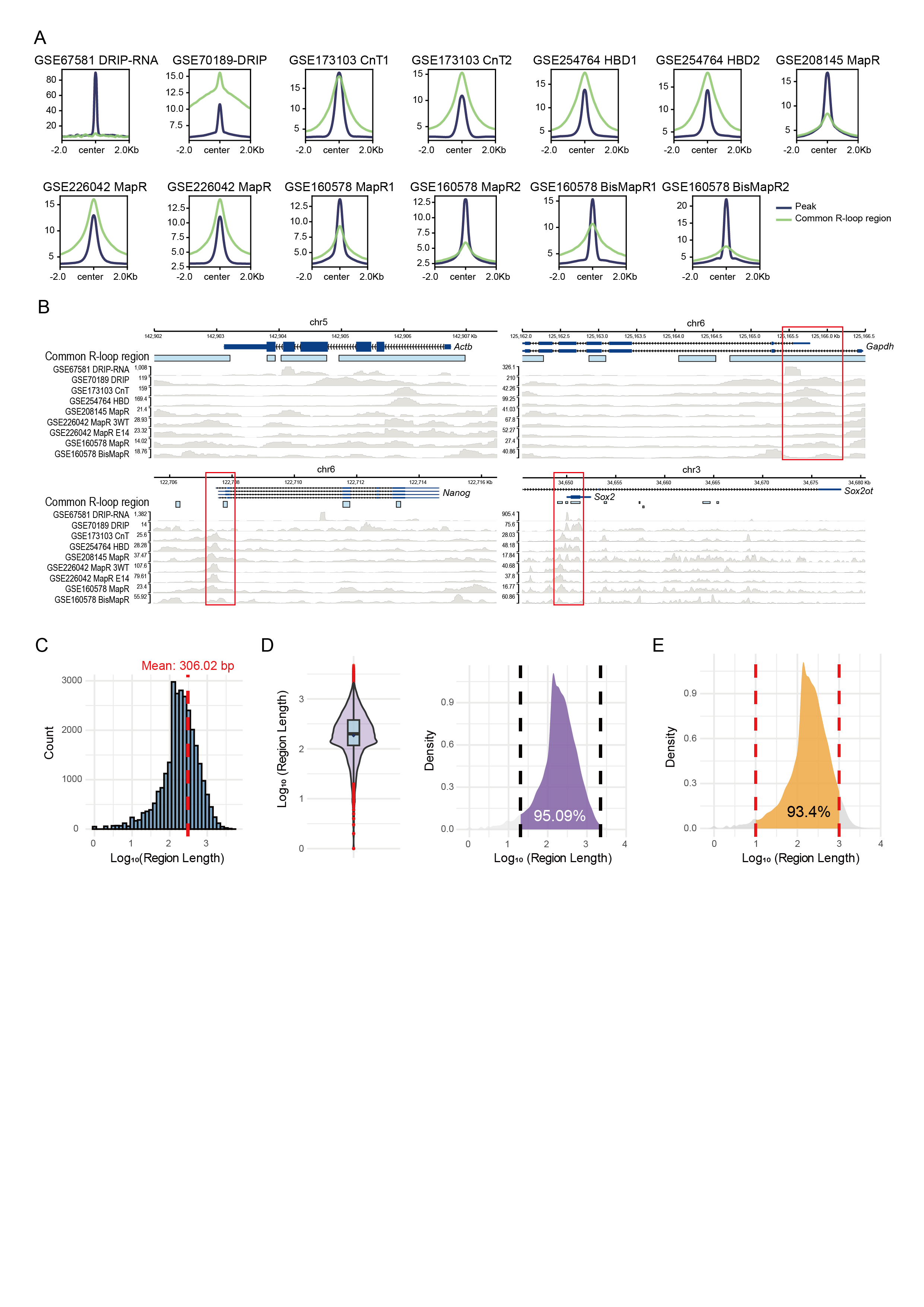

Supplement: Supplementary file 1 [file epigenomes-10-00016-s001.zip › Figure S1.png]

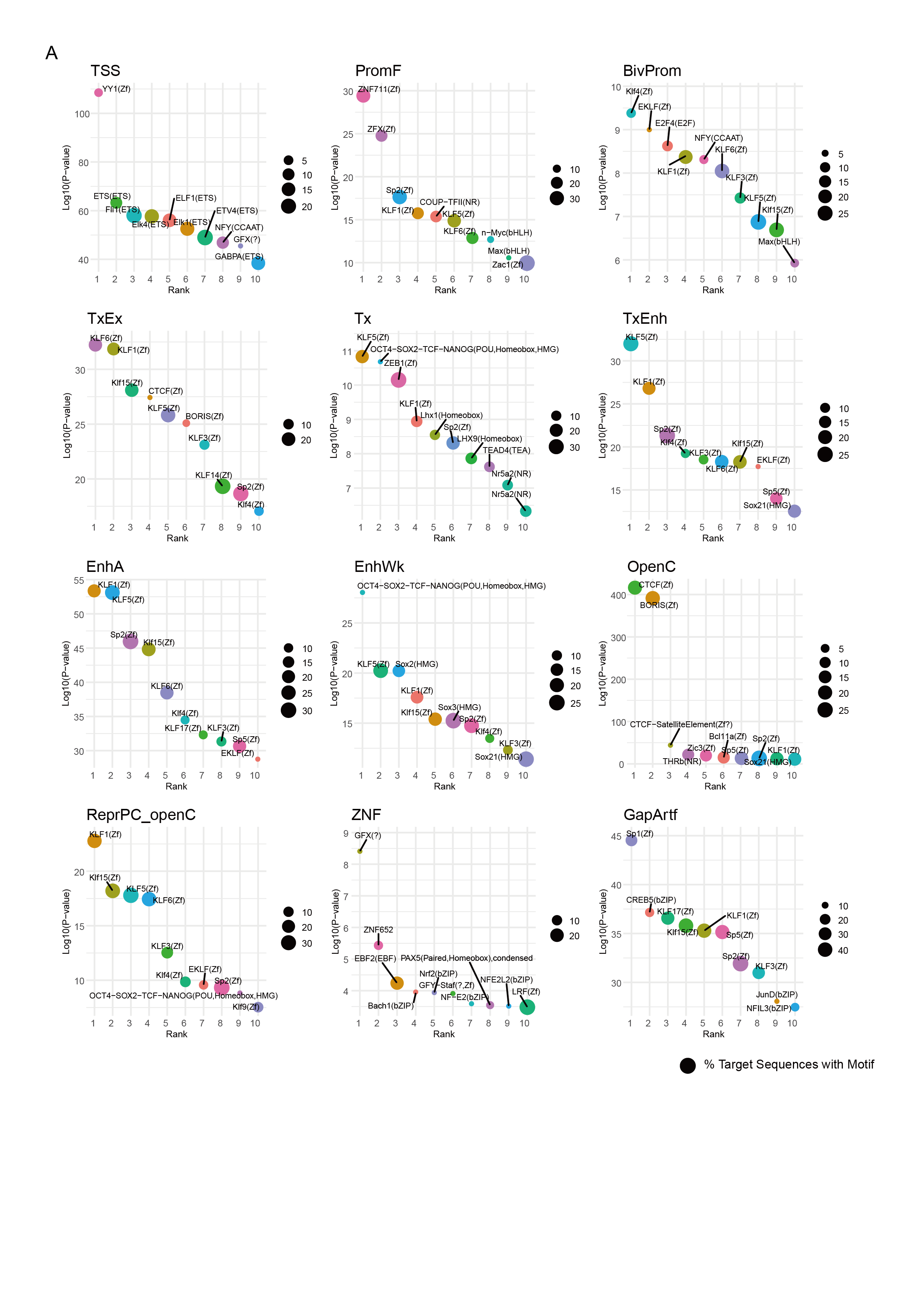

Supplement: Supplementary file 1 [file epigenomes-10-00016-s001.zip › Figure S10.png]

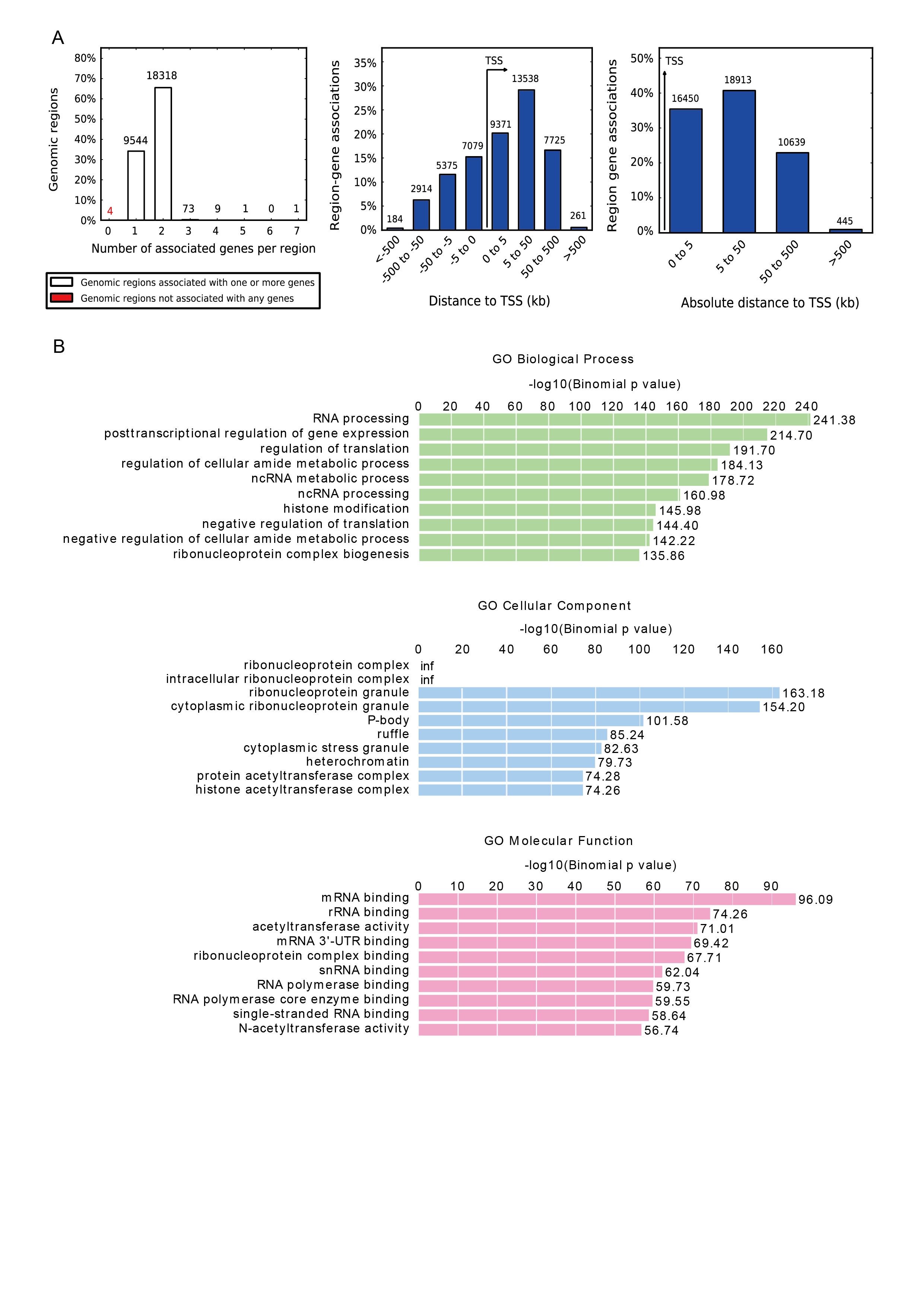

Supplement: Supplementary file 1 [file epigenomes-10-00016-s001.zip › Figure S2.png]

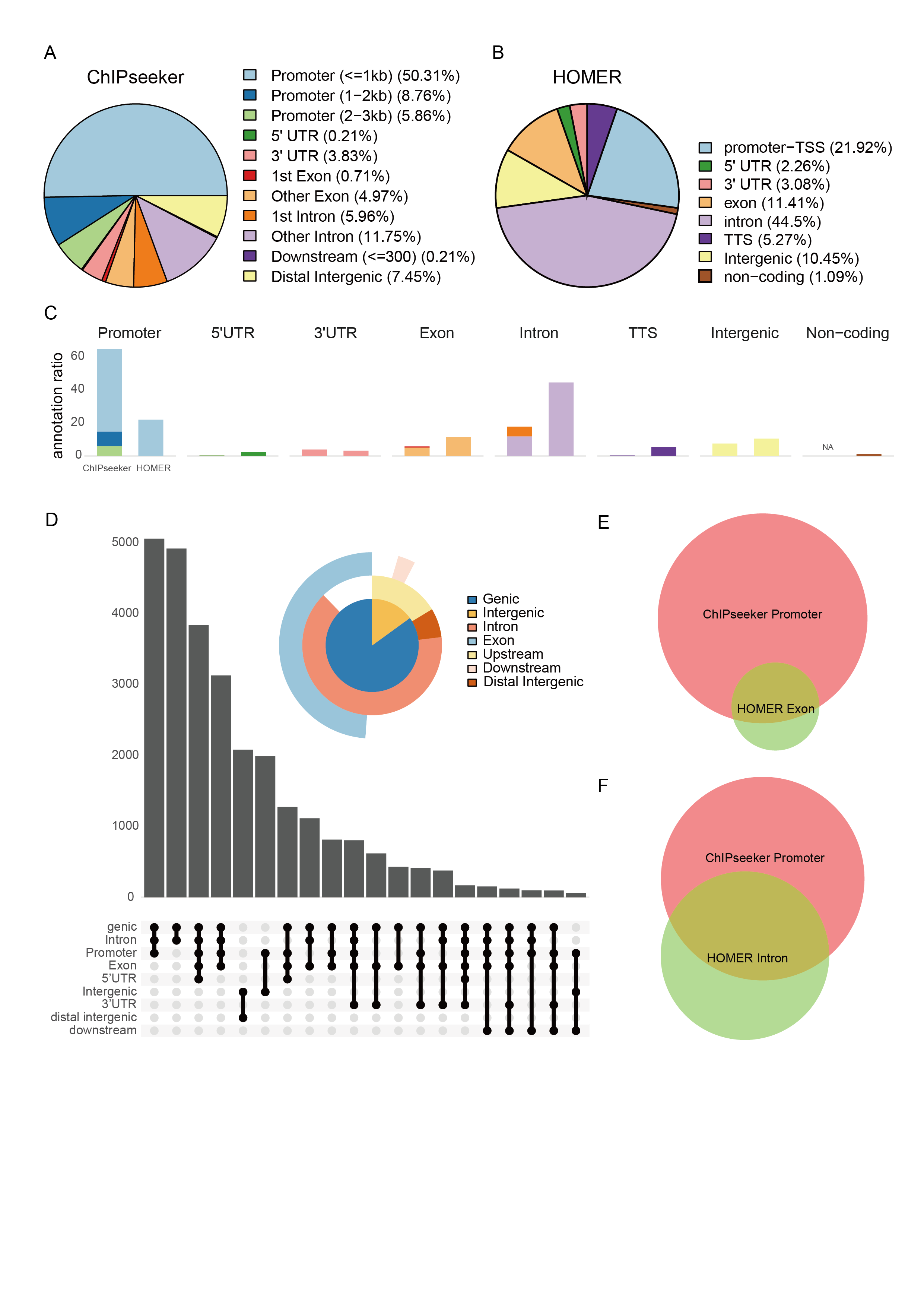

Supplement: Supplementary file 1 [file epigenomes-10-00016-s001.zip › Figure S3.png]

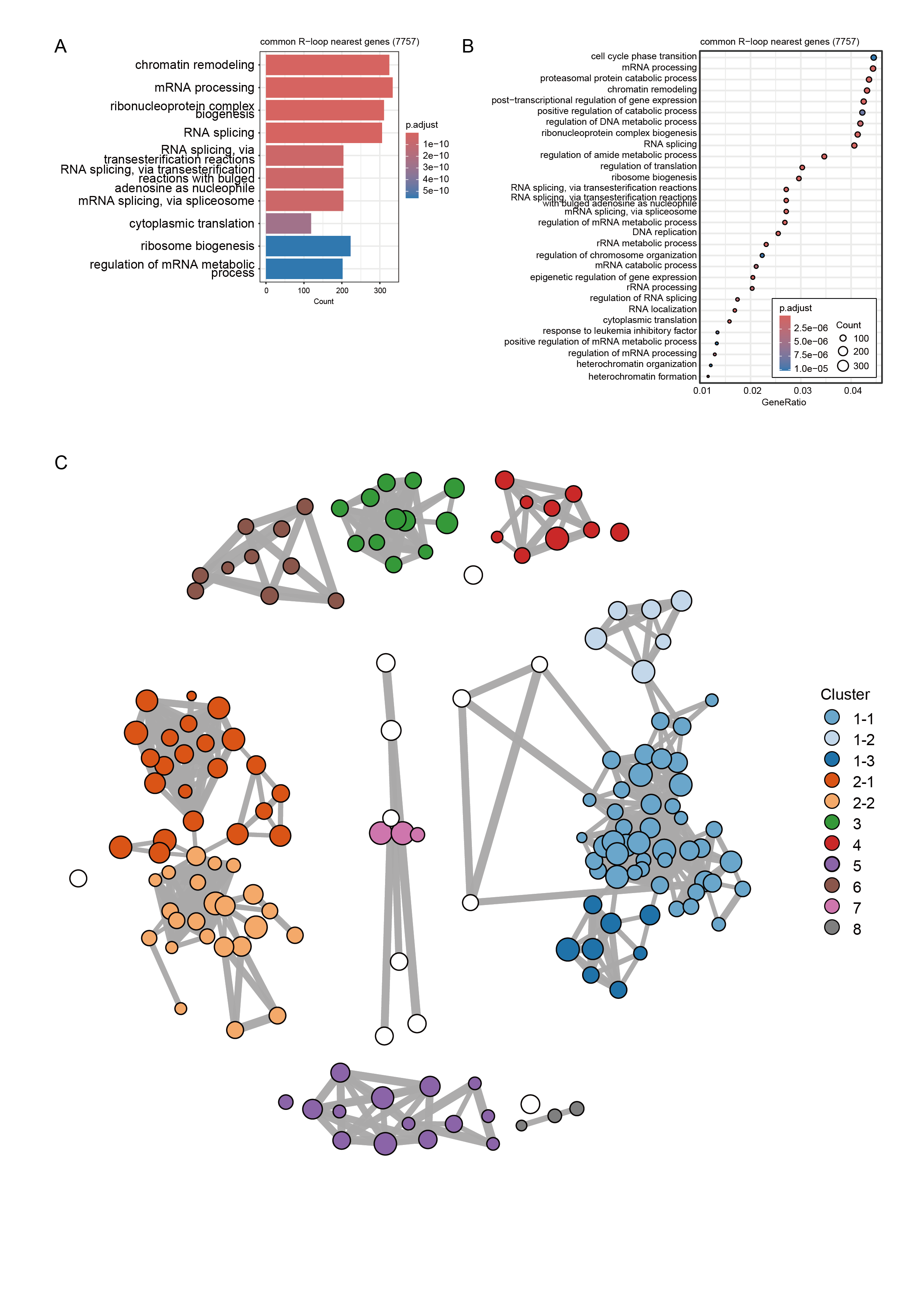

Supplement: Supplementary file 1 [file epigenomes-10-00016-s001.zip › Figure S4.png]

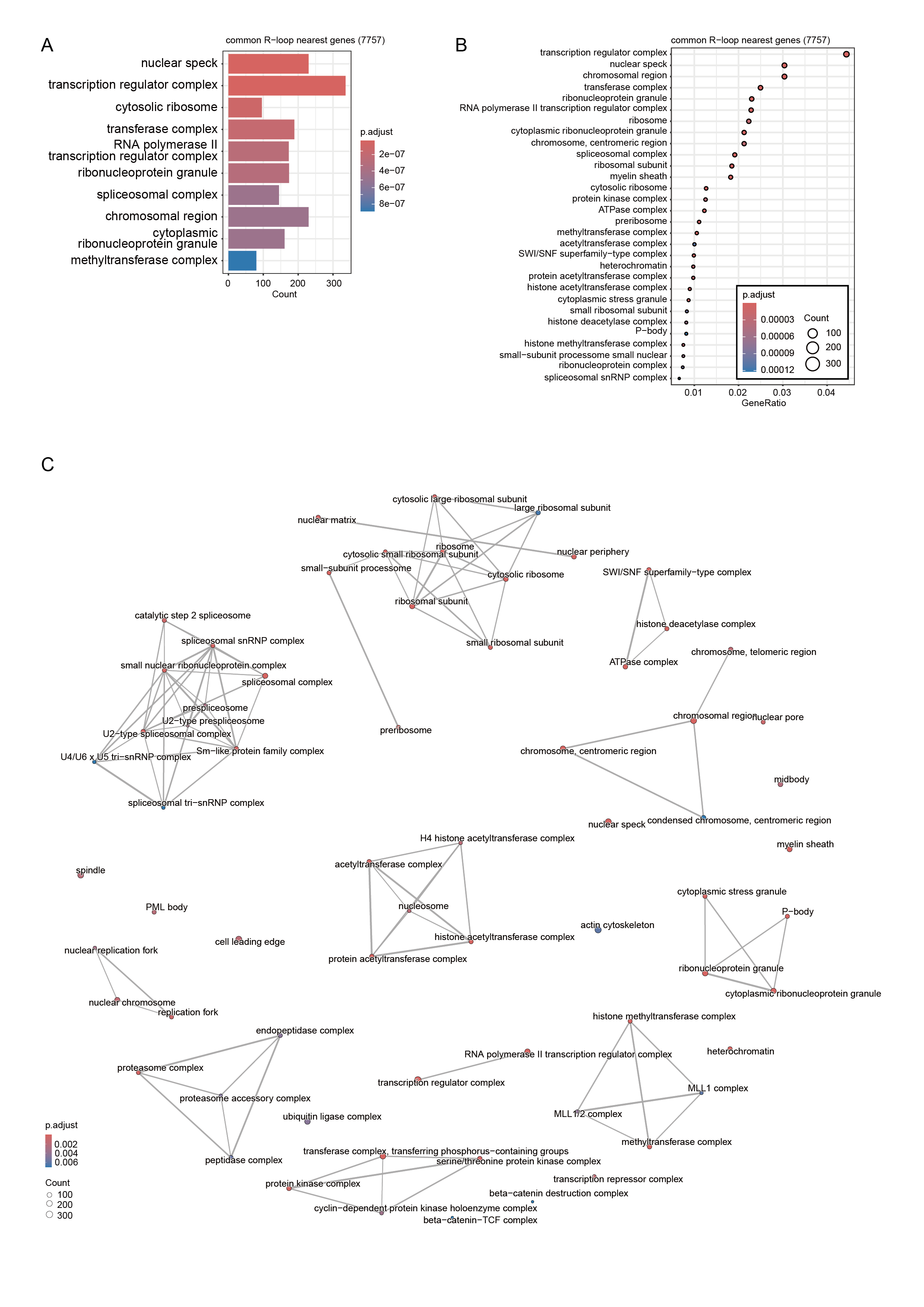

Supplement: Supplementary file 1 [file epigenomes-10-00016-s001.zip › Figure S5.png]

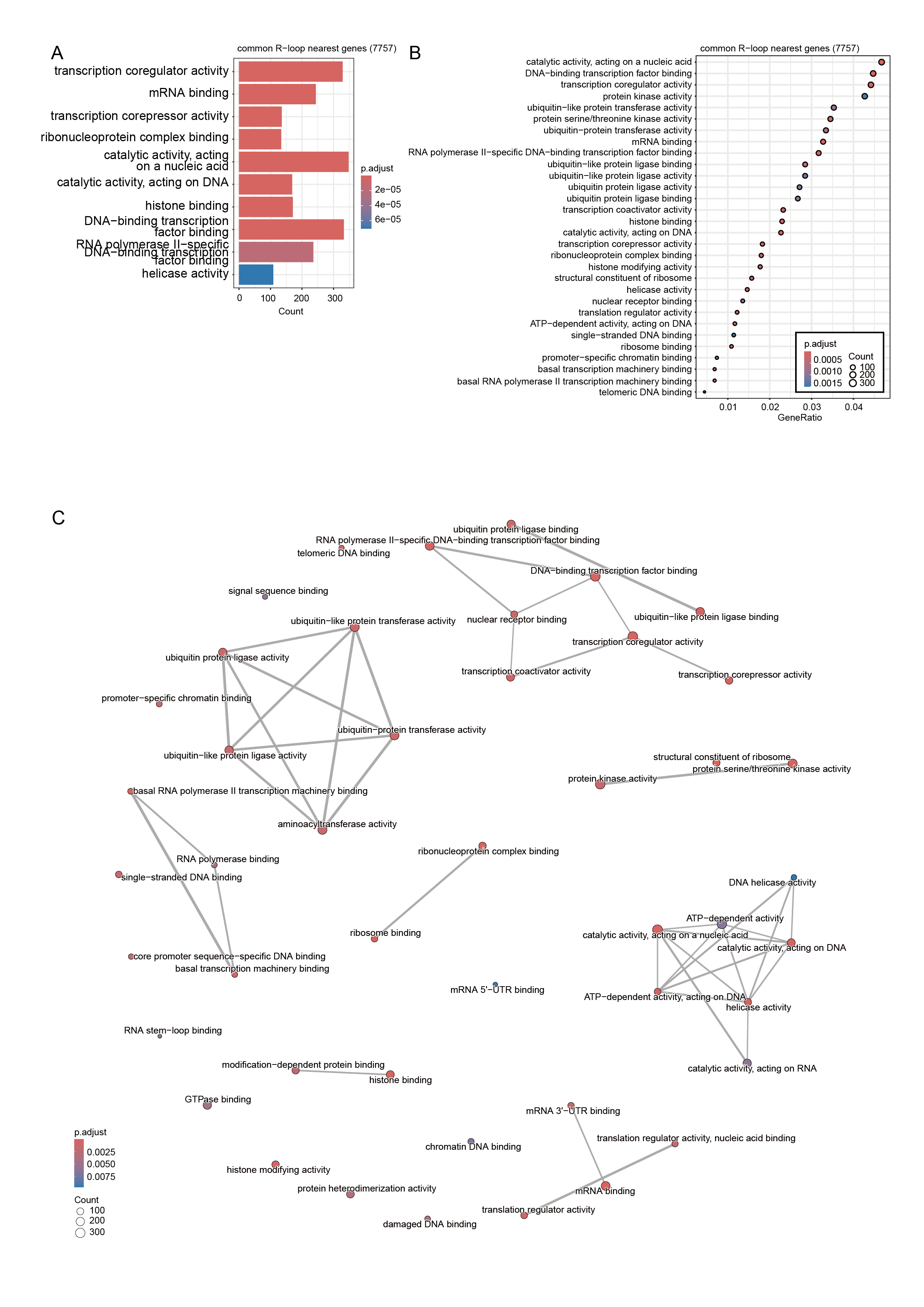

Supplement: Supplementary file 1 [file epigenomes-10-00016-s001.zip › Figure S6.png]

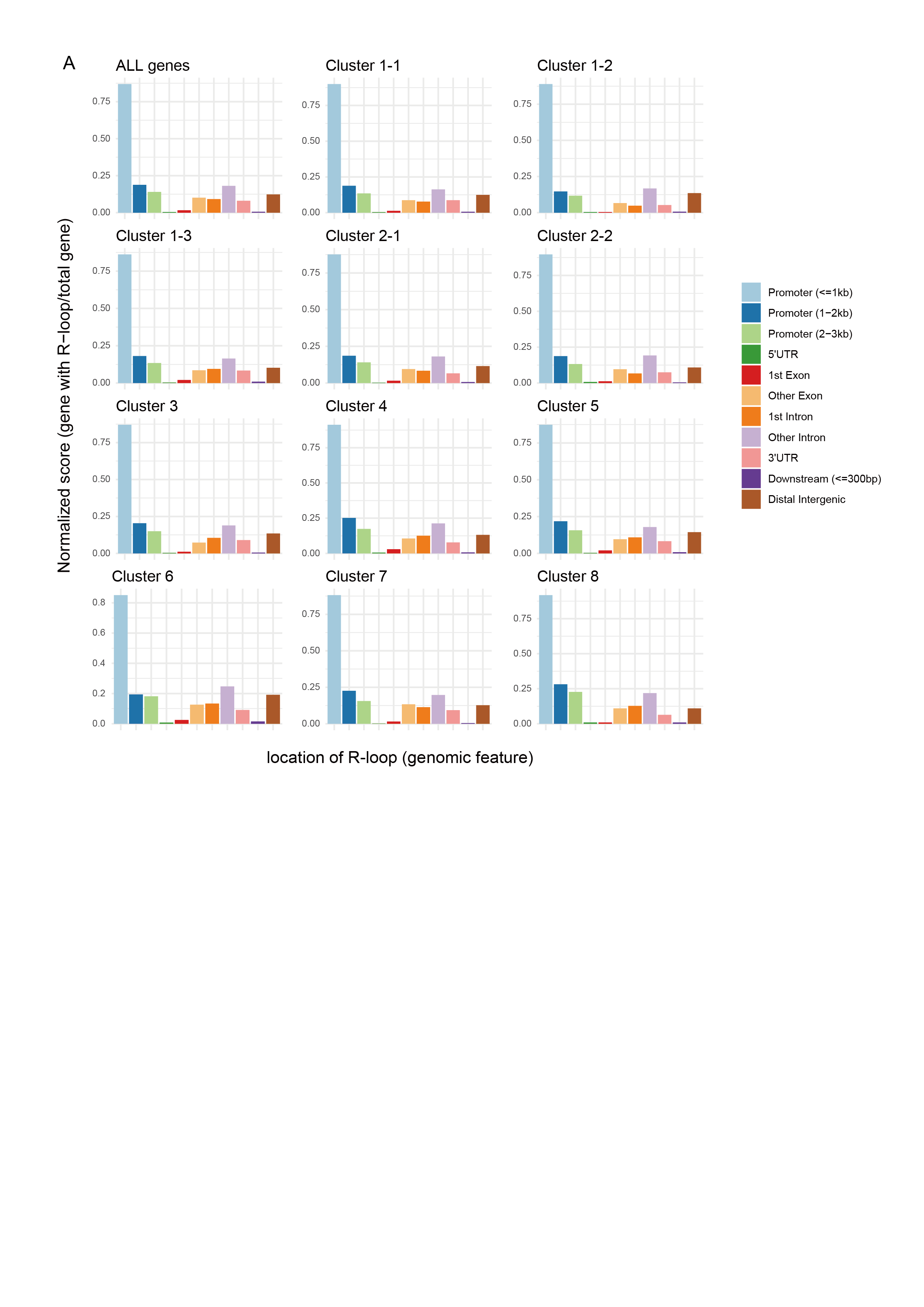

Supplement: Supplementary file 1 [file epigenomes-10-00016-s001.zip › Figure S7.png]

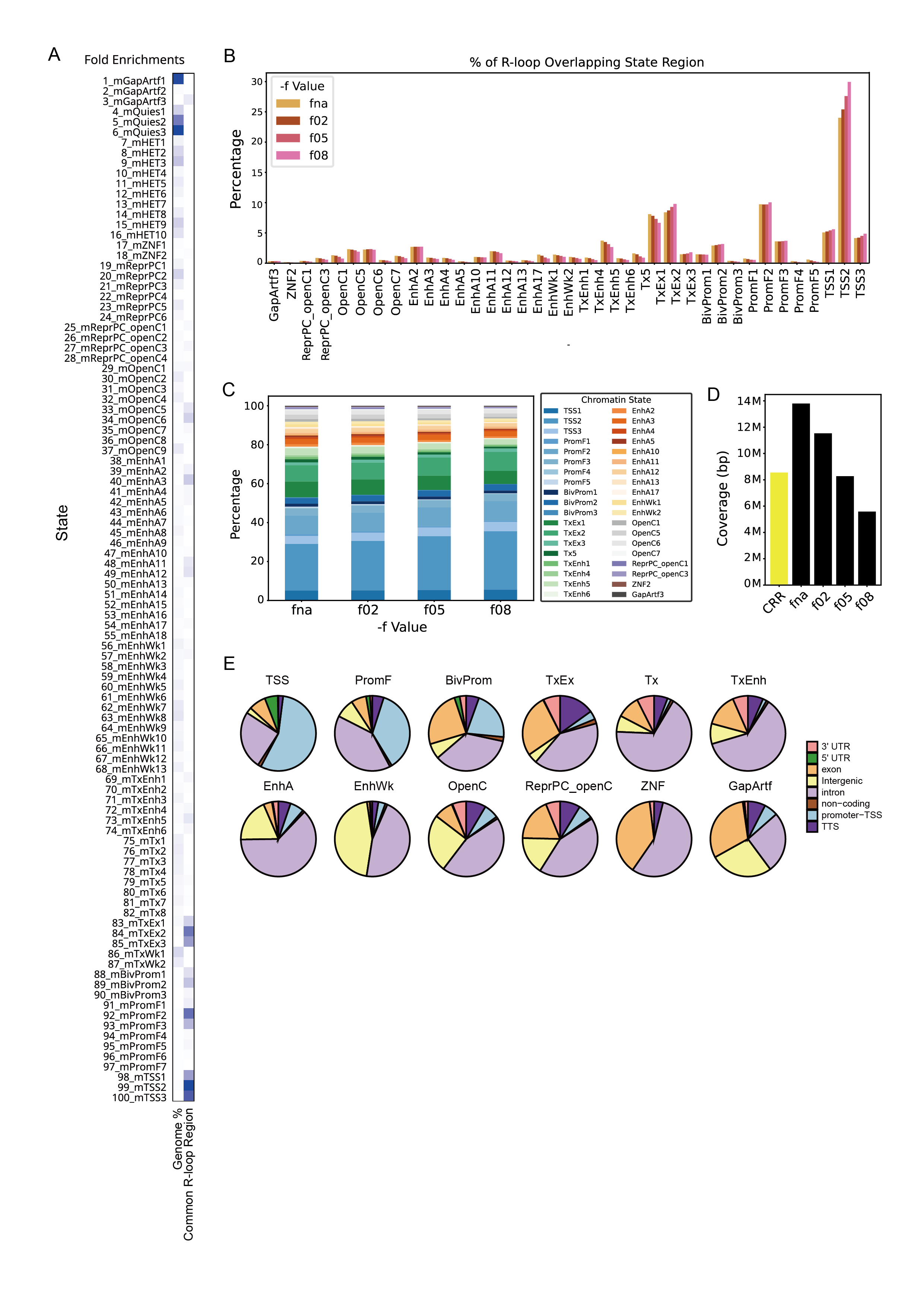

Supplement: Supplementary file 1 [file epigenomes-10-00016-s001.zip › Figure S8.png]

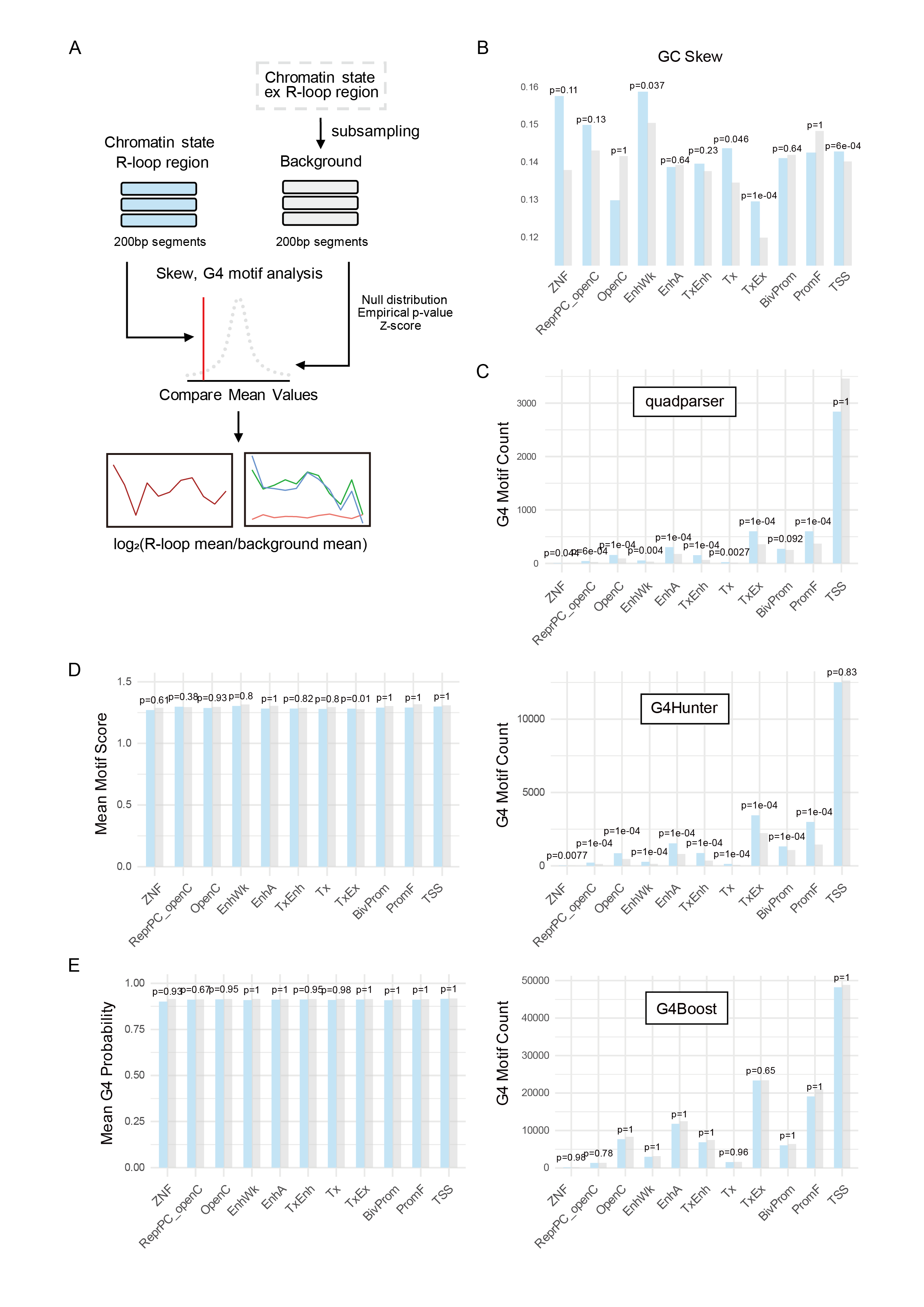

Supplement: Supplementary file 1 [file epigenomes-10-00016-s001.zip › Figure S9.png]
